# Research and Management of Rare Diseases in the COVID-19 Pandemic Era: Challenges and Countermeasures

**DOI:** 10.3389/fpubh.2021.640282

**Published:** 2021-04-15

**Authors:** Sanjana Fatema Chowdhury, Syed Muktadir Al Sium, Saeed Anwar

**Affiliations:** ^1^Department of Genetic Engineering and Biotechnology, School of Life Sciences, Shahjalal University of Science and Technology, Sylhet, Bangladesh; ^2^Child Health Research Foundation, Dhaka, Bangladesh; ^3^Department of Biotechnology, Agriculture Faculty, Bangladesh Agricultural University, Mymensingh, Bangladesh; ^4^Department of Medical Genetics, Faculty of Medicine & Dentistry, University of Alberta, Edmonton, AB, Canada

**Keywords:** COVID-19, rare disease, clinical management, counseling, telemedicine

## Abstract

The ongoing coronavirus disease 2019 (COVID-19) pandemic has disrupted every aspect of our life. The need to provide high-level care for an enormous number of patients with COVID-19 infection during this pandemic has impacted resourcing for and restricted the routine care of all non-COVID-19 conditions. Since the beginning of the pandemic, the people living with rare disorders, who represent a marginalized group of the population even in a normal world, have not received enough attention that they deserve. Due to the pandemic situation, they have experienced (and experiencing) an extreme inadequacy of regular clinical services, counseling, and therapies they need, which have made their life more vulnerable and feel more marginalized. Besides, the clinicians, researchers, and scientists working on rare genetic diseases face extra challenges due to the pandemic. Many ongoing research projects and clinical trials for rare and genetic diseases were stalled to avoid patients' and research staff's transmission to COVID-19. Still, with all the odds, telehealth and virtual consultations for rare disease patients have shown hope. The clinical, organizational, and economic challenges faced by institutions, patients, their families, and the caregivers during the pandemic indicate the importance of ensuring continuity of care in managing rare diseases, including adequate diagnostics and priority management strategies for emergencies. In this review, we endeavored to shed light on the issues the rare disease community faces during the pandemic and the adaptations that could help the rare disease community to better sustain in the coming days.

## Backgrounds

The coronavirus disease 2019 (COVID-19) pandemic remains an enormous global challenge due to its persistent spread and unpredictable disease course. As of February 2021, the disease has caused ~110 million confirmed cases and ~2.5 million deaths (1). Current understanding of the COVID-19 pathobiology indicates that infection with severe acute respiratory syndrome coronavirus 2 (SARS-CoV-2), the etiologic cause of COVID-19, results in an impaired adaptive host inflammatory response, causing excessive activation of innate pathways to generate a cytokine storm and edema leading to pulmonary fibrosis and severe pathology (2, 3). Risk factors for adverse outcomes include old age, male sex, and comorbidities (4, 5). Also, people with weakened immune systems face a higher risk. With the great efforts of clinicians, researchers, and academicians worldwide, vaccines have rolled out for mass vaccination in some countries, and other countries are also in the process of starting vaccination programs. The world is hoping to get back to a “normal” world soon. However, there is still uncertainty of management strategies for the patients who require critical care and effective treatment. Researchers and clinicians have so far recorded only a dearth of reports of infected patients with rare diseases. In the literature and our own experience, few patients with rare diseases have presented COVID-19, perhaps because of their awareness of risks and preventive measures (6, 7). As a result, only a few small cohort studies and case reports on the effects of COVID-19 on people with rare diseases, e.g., thalassemia, are available (8–10). Because of the insufficient clinical evidence, any comment on the relationship between certain rare diseases and COVID-19 may be regarded as mere theories; however, they should not be ignored.

The COVID-19 pandemic has heightened uncertainty over all aspects of our life, including family and community life, economies, and healthcare, and none more so than the most vulnerable of us—individuals with rare diseases. There are between five to eight thousand rare diseases, most of them with a genetic basis, affecting ~400 million people worldwide (11–13). Even in the best of times, people with rare diseases and their caregivers report significant care inadequacies and unmet clinical needs. Besides, the difficulty and expense of assembling large cohorts of affected individuals for study and garnering research funding is already a concern for researchers. Along with the general anxieties about health concerns everyone else has, people with rare diseases have a double burden of challenges due to the pandemic. They also face uncertainty about the supply of medications and the accessibility of essential occupational therapies they need regularly.

The COVID-19 pandemic has also impacted clinical and health research severely. It caused stall many translational, clinical, and basic science research (14), thus influencing every medical practice aspect. It has also led to a sudden rift in the medical research on diseases other than COVID-19, making the rare disease research more challenging and slower. Numerous experiments and clinical trials have been abandoned, suspended, or post-poned (15, 16). Many have paused on their ongoing clinical research to focus on SARS-CoV-2 related research or made substantial modifications to ensure safe clinical care in the hospital. As a result, the research and development on other diseases, e.g., cancer, cardiovascular conditions, and rare diseases, may experience (and already is experiencing) disruption—potentially causing the people living with these diseases to suffer delayed access to new drugs and/or management strategies (17). While combatting the pandemic mainly focusing on the general people, collaborations between the patient, scientific communities, government, diagnostic service providers, and rare disease research need prioritization to ensure proper management of rare diseases. Persisting needs include dissemination of specific knowledge regarding optimal care and research to prevent, treat, and cure disease.

This review discusses the difficulties and struggles of rare disease patients, caregivers, and researchers studying such diseases, amidst COVID-19 and even after the pandemic is over. Also, it focuses on how to manage these challenges better in a world free of COVID-19.

## Impact of COVID-19 on Rare Disease Communities

**Figure 1:**
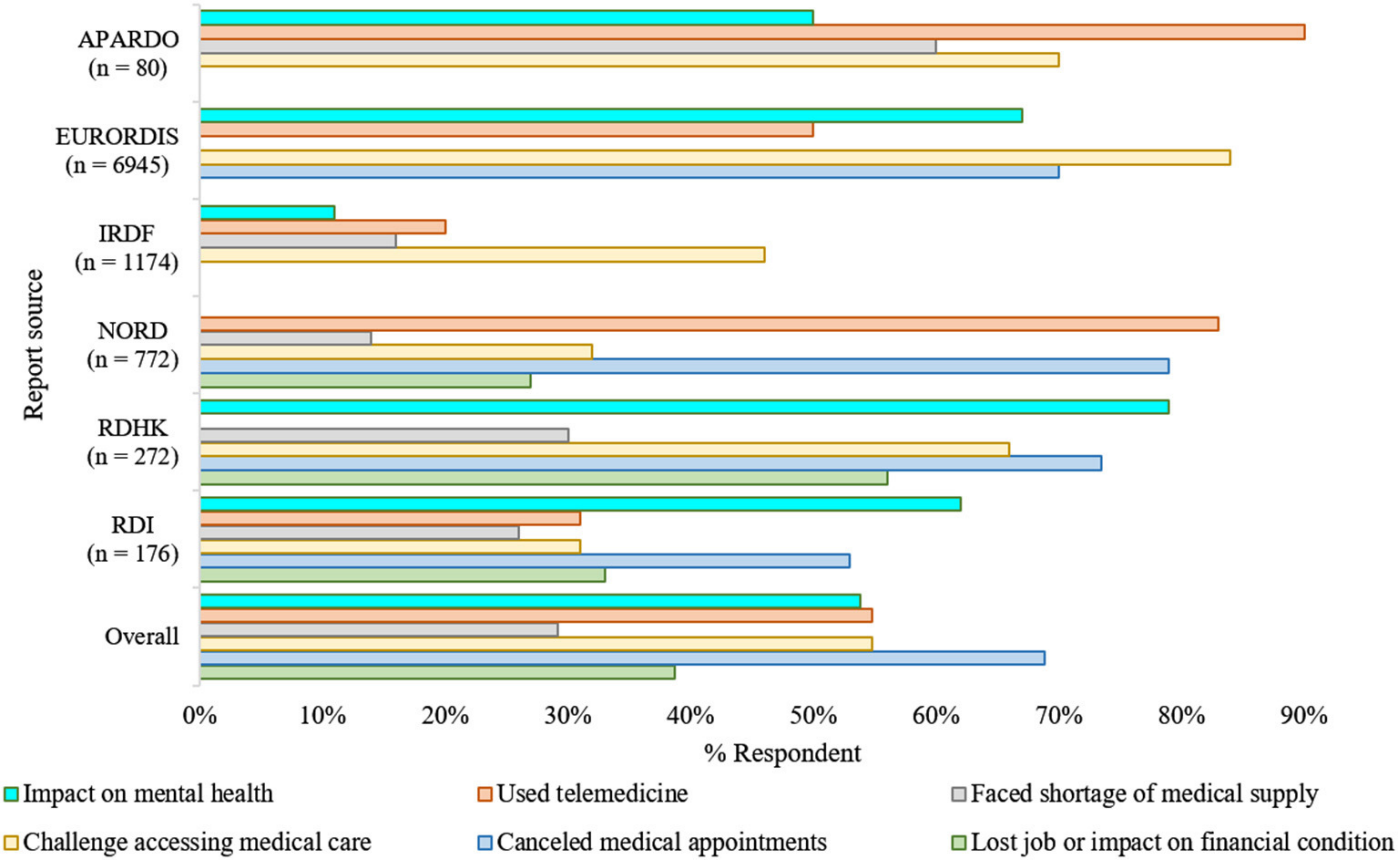
The impacts of COVID-19 in the rare disease community (7, 18–22). Overall, as adjusted estimation from multiple surveys reflects, the pandemic's unexpected emergence had a substantial repercussion on the regular healthcare, mental health, and financial arrangements of the rare disease communities. The consequences were asymmetric in various aspects; however, they were analogous irrespective of the geographical region where the surveys took place (7, 18–23). NORD, National Organization of Rare Disease; EURORDIS, European Organization for Rare Diseases; RDHK, Rare Disease Hong Kong; IRDF, Italian Rare Disease Foundation; RDI, Rare Disease Ireland; APARDO, Asia Pacific Alliance of Rare Disease Organizations. The surveys included were independent of each other and not all focused on the same parameters.

The far-reaching impacts of the COVID-19 pandemic on rare disease communities were reflected in a recent report by the U.S. National Organization for Rare Disorders (NORD) (18). The report suggests that almost all respondents (~98%) were overwhelmingly concerned and worried about the pandemic due to several reasons (Figure 1). Among them, 95% of families had been directly influenced by COVID-19, with more than 50% having medical appointments replaced with a telephone or video call. Besides, three out of every five respondents expressed concerns about a potential shortage of medication and medical supplies.

The COVID-19 pandemic led to a loss of jobs, whether temporarily or permanently, for over one-fourth of the respondents. Over 10% of these job losses resulted in a loss of their health insurance (24). As many individuals with rare diseases require continuous treatment support, which needs the families' financial stability, job loss due to pandemic has directly impacted their routine management.

The European Organization for Rare Diseases (EURORDIS) found a similar impact of the COVID-19 pandemic on people living with rare diseases. It reported that nine out of ten Europeans living with a rare disease had faced pause or interruptions in their regular health care since the beginning of the pandemic, and most of them were worried that this pause could be life-threatening. Most pre-scheduled surgeries, transplants, and rehabilitation therapies, e.g., speech and physical therapies, have been canceled or postponed (19). As the pandemic persists, some hospitals have temporarily closed rare disease units, and as a result, patients who used to receive treatments in these units are experiencing difficulties.

For the time being, most consultants are now trying to provide support and services to people with rare diseases by telephone, videoconferencing. It was reported that nearly half of the respondents had received telemedicine service as in-person consultations are now not recommended. In addition, according to the survey of EURORDIS, most of the respondents have no or limited access to medical therapies such as chemotherapy, infusions, and hormonal treatment. Moreover, diagnosis assessments, e.g., blood or cardiac tests and medical imaging are important parts of daily care for such individuals; however, more than half of the respondents no longer have access to diagnostic facilities due to lockdown and fear of virus transmission. Else, appointments, follow-up meetings are mostly on post-pone, regular therapy schedules are interrupted, and urgent visits are hindered.

The pandemic had significant and enormous repercussions on the healthcare systems as they went through a drastic reorganization to respond to this health emergency (25). Rare disease communities worldwide are particularly impacted due to these reorganizations, especially in terms of their regular healthcare (Figure 1). Studies led by UNIAMO Italian Rare Disease Foundation (UNIAMO–Federazione Italiana Malattie Rare onlus) and Rare Disease Ireland report similar outcomes (20, 21). Ninety five percentage (*N* = 1,174) of the respondents from Italy reported having rare conditions, 14% of whom had two or more pathological conditions, and ~1% had a condition without confirmed diagnoses (20). Over half of the participants (52%) from Italy, one of the hardest-hit countries by the pandemic, indicated that they had given up hospital treatment to help limit their infection exposure (20). Another half (46%) faced problems in continuing their ongoing medication/therapies, as the government forced the outpatient facilities to ramp down in their service to operate only for life-saving and urgent interventions (20). In the Rare Disease Ireland survey, 53% of the participants reported cancellation of a scheduled medical appointment at a cost to the immediate and long-term health and well-being of those living with a rare condition (21). Also, 26% of these respondents reported difficulties in accessing medicines and other medical supplies. Besides, 62% believe that COVID-19 is hurting their mental health. Similar findings were reported by the Rare Disease Hong Kong (RDHK); more than 50% of the study cohort, consisting of 272 participants with 89 distinct rare conditions, opined that their medical treatment was interrupted by pandemic (7). Many participants also complained about deficits in the healthcare provision, shortage of medical supplies, and mental instability during this period (7, 26). Studying participant responses from 10 different countries affected differently by the pandemic, the report from Asia Pacific Alliance of Rare Disease Organizations (APARDO) almost recapitulated the surveys from Italy, Ireland, and Hong Kong (22).

## Challenges to Patients

The novel coronavirus disease COVID-19 possesses challenges for millions of people with rare diseases, from possible increased anxiety and stress to potentially reduced access to necessary medical treatment. Besides, some pathologies lead to the greater fragility of the rare disease patient, such as immune deficiencies, complex congenital syndromes, chronic lung diseases, congenital heart disease, and hereditary metabolic pathologies at risk of acute decompensation. Therefore, many patients with rare diseases generally require ongoing assistance, from drug therapies to rehabilitation treatments to medical devices, often lifesaving.

Most of the rare disease patients have specific pathologies linked to increased perception of the risk of possible side effects following SARS-CoV-2 infection. Favism, for example, is a rare disease caused by Glucose-6-phosphate dehydrogenase (G6PD) deficiency and G6PD deficient cells are more vulnerable to SARS-CoV-2 infection. G6PD enzyme is sensitive to oxidative action on red blood cells, potentially triggering hemolytic crises. Among the administered drugs to deal with the pandemic from SARS-CoV-2, chloroquine and hydroxychloroquine have oxidative properties, triggering severe hemolysis in favism patients (27, 28). However, data from multiple rare connective tissue disorder patient registries suggest that anti-rheumatic drugs, e.g., hydroxychloroquine is impartial, prolonged use of corticosteroids at moderate to high could be deleterious and the use of some specific TNF inhibitors could produce protective outcomes (29, 30). In addition, many autoimmune or neuromuscular diseases can be treated with cortisone or immunosuppressants that determine an increased risk, both in terms of morbidity and mortality, in case of respiratory virus infection, such as SARS-CoV-2 infection (31). Interestingly, rare connective tissue disorders and immune-compromised rheumatic disease patients were not found to be at a higher risk for SARS-CoV-2 infection (6, 31, 32).

However, the challenges in the management of rare diseases are three-fold compared to diagnosing and treating common diseases (33). They may struggle to find appropriate physicians knowledgeable about the disease's pathophysiology, the natural course of the disease, and epidemiological information to manage them (12). Also, many of the individuals with rare diseases may struggle to receive an early diagnosis and suffer the consequences. For instance, a newborn with a rare condition may experience proper and delayed diagnosis under the current situation, which may significantly add to its sufferings in the coming days. Besides, this can potentially result in a rise in the cost needed for disease-specific treatment (34, 35).

The National Institutes of Health (NIH) estimates that only 5% of rare diseases have approved treatments (36, 37), while many therapies presumably work only at the young age of the patients, and if the disease is in primary stage (38). Many rare diseases are progressive, and the clinical condition deteriorates over time (39). As an immediate response to the pandemic, most pharma industries and researchers concentrate on therapy development for COVID-19, and it is causing a halt in the development of therapeutics for diseases other than COVID-19, including rare diseases. Since the outbreak, they are fighting without proper palliative care, presumably letting them down while fighting a progressive disease (40). Thus, for rare disease patients, such a pause in development is effectively a regression in progress.

## Challenges to Investigators Studying Rare Diseases

Investigators wishing to study the clinical progressions, pathomechanism, and natural history of rare diseases face significantly more obstacles than common disease researchers (33). For example, constituting a cohort of adequate size for a clinical study is a lot more difficult for rare disease investigators. It often requires international or multi-institutional collaboration. The COVID-19 pandemic situation has added to the impediments to gather such cohorts as effective collaborations have become tougher to develop.

Besides, funding support for rare disease research is usually limited (41). Since the pandemic began, scientists working on preclinical studies hoping that human trials could be launched by the coming year(s) had to shut down most of their experiments (42). Many of the rare disease researchers had to switch gear to facilitate more robust research focusing on COVID-19 (43). However, delays in producing a treatment could mean the forever loss of some people, maybe kids, who live with rare diseases, and some may progress to a non-recoverable or non-manageable state from where they could be treated.

## Challenges Related to Funding

The impact of COVID-19 on the currently ongoing research projects and funding was so crippling and will undoubtedly be long-lasting. Many organizations that usually fund research on rare diseases are now facing financial crises (44). NIH (45), Patient-Centered Outcomes Research Institute (PCORI) (46), and other major funders took prompt measures for making the proper guideline on proposal submission and fund distribution that allows grant personnel to be paid in a relaxed timeline. Research institutions prioritize COVID-19 related research proposals while other proposals are delayed or postponed (47). Also, governments are spending a considerable portion of their out-of-pocket budget to manage the COVID-19 situation. Many organizations are moving their money to start COVID-19 related works (48, 49).

The genetic sequence of SARS-CoV-2 was released in early January 2020, just weeks after the first reported cases, significantly accelerated research and therapeutic development on COVID-19. As of March 14th 2021, over 5,017 clinical trial studies related to COVID-19 are registered on ClinicalTrials.gov (50). After almost 5 months since the genetic sequence release, 148 studies associated with hydroxychloroquine, 13 with remdesivir, 50 with vaccines, and 100 with diagnostic testing were registered (51). Another 3,733 different studies are registered on the World Health Organization's International Clinical Trials Registry Platform (WHO ICTRP) (52).

Furthermore, as the world has recently seen a huge blow due to an infectious disease, we may observe a flow of money toward infectious disease research from non-communicable and rare disease research in the coming future (25, 53). In the long run, the pandemic will possibly force the reallocation of research grants at the expense of research areas funded before the pandemic.

## Supply of Medical Equipment and Therapies

Few human-derived rare disease therapies such as plasma, blood factors, and cell therapies are being studied as treatments for COVID-19 (54). Thus, they may be facing the risk of shortages. For example, Immunoglobulin (Ig), derived from human plasma, has a complex supply chain and is used to treat primary immune deficiency and others (55), has faced shortages in the US and some other parts of the world for some time (56). Some essential medical supplies have also faced dramatic price hikes during this period (57). Additionally, blood donations have significantly been reduced due to social distancing and heightened infection concerns (58). Those who are willing to donate blood are being screened strictly to avoid transmission and ensure safety protocols (59), which is also putting pressure on the already over-stretched systems.

In early 2020, hydroxychloroquine, a well-known drug for autoimmune disorders, e.g., lupus and rheumatoid arthritis, had gained some focus as a potential COVID-19 treatment (60), resulting in its place in the FDA's (Food and Drug Administration) shortage list for months. Similarly, as few companies are trying to develop plasma COVID-19 therapies (61), it is expected to put pressure on plasma supply. The FDA is working proactively to evaluate the entire supply chain, including active pharmaceutical ingredients, finished dose forms, and other components that may be impacted in any supply chain area due to the COVID-19 outbreak, along with pharmaceutical companies and manufacturers, including those for rare disease therapies.

## Clinical Trials During COVID-19

The effect of COVID-19 on clinical trial research has been enormous, with thousands of trials—around 80% of non-COVID-19 trials—being stopped or interrupted (62). The major difficulty for clinical trials lies in the in-person visits to hospitals or clinics for either follow-up or therapeutic administration. The rare disease patients have a higher risk of contracting the virus if the hospitals do not have separate areas for COVID-19 patients. Thus, many companies postponed or canceled new clinical trials and pushed back trial visits for existing ones (42). The National Cancer Center Singapore faced difficulties with more than 200 ongoing clinical trials due to travel restrictions from different countries, as many participants come from the South Asian region (42). In a report from Spain, the La Paz University Hospital had 59 hemophilia-related clinical trials and registries active in the Thrombosis and Homeostasis Unit, which was interrupted due to a nationwide lockdown (63). However, they tried to mitigate this situation through a telemedicine program, which eventually proved to be partly able to replace in-person patient care (63, 64).

Moreover, clinical investigators responsible for clinical trials are being reallocated to manage a significantly higher number of COVID-19 patients. Many clinicians, scientists, research administrators, clinical trial-related officials were pulled away from working on clinical trials to work in emergency medical care, especially during the first months of the pandemic (62). They are, in most cases, yet to resume from where they stopped. Moreover, clinical research administrators responsible for clinical trials are being reallocated to manage a significantly higher number of COVID-19 patients in clinical setups and COVID-19 related clinical trial programs. These represent significant challenges in maintaining clinical trial continuity in the coming future. In addition, the ramp down or cancellation of trials will have a superfluous effect on early career researchers, and even those who may be able to work from home—biostatisticians and epidemiologists—suffer the equivalent challenges that many have in maintaining work-life balance, which is especially true for those with kids (62).

## COVID-19 Vaccination and Rare Disease Community

The FDA granted emergency use authorization of two COVID-19 vaccines, the Pfizer/BioNTech vaccine and the Moderna vaccine (65), last December 2020. To date, millions of people worldwide have been receiving the vaccine doses (66). Scepticisms over the vaccines' efficacy due to emergency use authorizations are on the discussion; this concern is heightened among the rare disease communities as there were not enough rare disease individuals for the clinical trial. Some are also hoping to get genetic therapy after getting vaccinated, putting them into concerns over the effects of vaccination. Nevertheless, the officials of the regulatory boards have denied such speculations (67).

The Pfizer/BioNTech vaccine, which showed 95% efficacy (68) against COVID-19, had 43,548 people in the phase III trial (69), consisting of more than 2,900 people with chronic pulmonary disease (70). Still, none of the participants showed pulmonary hypertension (a rare condition). In comparison, the Moderna vaccine showed 94.1% efficacy in phase III clinical trial, which enrolled ~5% of the 30,000 participants with significant cardiac disease and pulmonary hypertension (70, 71). In addition, the COVID-19 mRNA vaccines exclusively target the SARS-CoV-2 virus and are unable to alter the recipient's genetic information (72, 73).

Also, people with rare diseases undergoing or expecting gene therapies are concerned if the vaccines are compatible with the therapy. Some gene therapies for rare diseases are based on adeno-associated viruses (AAV); however, that is a different virus that shares little similarity with coronavirus or vaccines. Some vaccines, e.g., the Oxford-AstraZeneca and CanSino vaccines, use adenovirus; however, these are completely different viruses from the AAV used for the gene therapies, despite the similar name (74). Nevertheless, rare disease patients undergoing or awaiting gene therapies, immunosuppressant drugs, blood-thinning medicines, or immunocompromised individuals are recommended to discuss with their clinicians to determine whether/when a vaccine is permitted.

## Emerging Complications During the Pandemic

As COVID-19 continued to spread, clinicians' concern was complications associated with SARS-CoV-2 in rare disease patients. Verdoni et al. reported ten cases of a Kawasaki-like disease in young boys and girls in Bergamo, Italy (75) from February 18 to April 20, 2020, i.e., during the peak of the pandemic in the country. It is a rare acute vasculitis that affects children under 5 years of age, and the coronary artery inflames throughout the body (76). Among the ten cases, two children had a positive PCR swab, and eight had a positive serology test for SARS-CoV-2. However, these tests' clinical relevance is unclear as they were not done at the same time. Most Kawasaki disease patients respond well to intravenous immunoglobulin, though 10–20% need supplementary anti-inflammatory treatment (77). In this cohort, eight children among ten received corticosteroids in high dose, in addition to intravenous immunoglobulin. These differences raised confusion, whether the cohort has Kawasaki disease with SARS-CoV-2 or an emerging Kawasaki-like disease is characterized by multisystem inflammation. Moreover, researchers have reported clusters of similar cases across Europe (78). In addition, patients with rare hematological disorders (79), especially sickle cell disease patients, are at higher risk of bacterial infections partly due to asplenic conditions (80). There is a chance that such bacterial infection may be misdiagnosed as COVID-19 infection and can delay access to life-saving antibiotics due to unnecessary isolation and panic (81). A study on 211 non-ICU COVID-19 patients showed that preexisting pulmonary hypertension (PH) and right ventricular dysfunction (RVD) were associated with severe outcomes in COVID-19 (82). Also, COVID-19 can result in neurological complications, e.g., rare encephalitis diseases and Creutzfeldt-Jakob disease, as the virus was reported to be identifiable in the cerebrospinal fluid (CSF) (23, 83).

While these rare and sporadic incidences may reflect pure coincidence, these undoubtedly bring extra concerns for the people living with rare diseases. Similarly, patients with cancer face severe bacterial infection risk due to vulnerable physical conditions (84). Late diagnosis of such conditions in the first pandemic wave shows how rare and difficult it is to recognize the disease in a deficient or malfunctioning healthcare system, which should be reorganized to deal with future pandemics. Moreover, studying the association between COVID-19 and rare diseases potentially provide important insights into physiological conditions that can be extended to understanding rare diseases and other relevant conditions.

## Adaptation of Rare Disease Research With the New Normal

**Figure 2:**
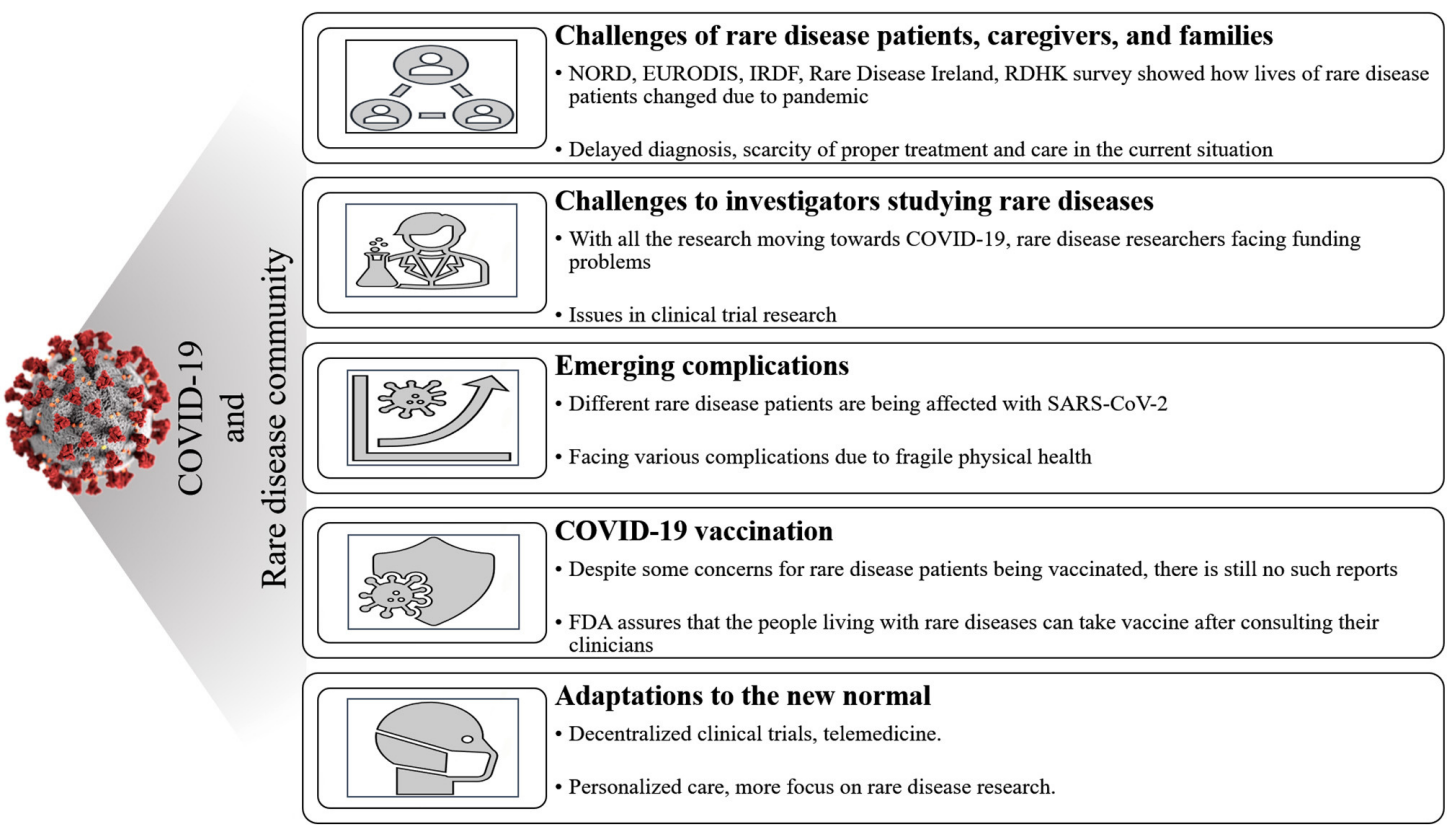
Summary of the impacts of the COVID-19 pandemic on the rare disease communities. The patients, stakeholders, investigators are under serious strain owing to the required care for patients with COVID-19, which has had a knock-on effect on the management of other patients, including the patients living with rare diseases. The pandemic interrupted the regular healthcare for people living with rare diseases and research focusing on rare diseases, mostly because of the extraordinary restrictions imposed to prevent and control SARS-CoV-2 transmission. Besides, many rare disease patients with fragile health are affected by SARS-CoV-2, putting them in a more challenging state. Besides, the fear of possible adverse effects of the vaccines on the management and treatment of rare diseases has been raised. The adaptations to the “new normal” and changes to clinical protocols to help prevent or control SARS-CoV-2 transmission (and any other outbreaks coming in the future) have also been a concern among the rare disease communities and are a point of discussion.

As the pandemic continues, the world has seen some ups and downs in terms of cases and fatality rates. The easing of public restrictions has resulted in a second wave. New cases are increasing since early August, which may carry with a lot of newer restrictions (17). Thus, countries need to be prepared for what is coming in the next winter. The government should engage vulnerable patients, including the rare disease patients, widely for essential health services. The unprecedented impacts of COVID-19 on the people living with rare diseases, their family and caregivers, researchers, and stakeholders (Figure 2) should be considered to avoid further damage.

COVID-19 pandemic has resulted in an unexpected economic downturn, affecting emerging biotech companies to survive and thrive amidst new safety guidelines and restructured core business strategies. They are applying for emergency capital to maintain continuity and push forward. While maintaining social distancing guidelines, biotech businesses transfer all medical research to exempt commercial collaborators from closed academic labs. Moreover, works that can be done using digital facilities, like conducting online meetings, data entry, and online data analysis, are being done online to reduce transmission.

The decentralization programs have been prioritized for uninterrupted clinical trials during the pandemic. Orphan drug developers and their partners are reshaping clinical trial administration, either entirely virtual or hybrid approaches (85), to adapt to the new normal. The FDA has also prepared flexible guidelines for clinical trials that allow the research to introduce virtual interviews or visits, self-administration, and remote monitoring. These changes will take time to cope up with the patients, caregivers, and even the clinicians. To make sure this works, companies will need to work with disease stakeholders, regulators, and everyone else in the health sector to design functional trials with successful results and ensure robust and standardized data collection (54).

Nonprofits rely heavily on in-person engagements to ensure continuity and must rethink how they raise money to maintain their work. Their fundraising strategies have been significantly restructured overnight. Without the in-person fundraiser events, non-profit companies emphasize funding from the virtual realm and corporate sources to keep going in a post-COVID-19 world. Once society returns to the new normal, research and development should move forward with creative and insightful ideas.

After the pandemic is over, our lives may not be the old normal again for an extended time. The general medical practice will be changed for an extended period, with social distancing and work from home. For rare disease patients, this would be a more crucial period than ever. Governments should focus on telemedicine services at this time to maintain social distancing. Typically, a fragile, rare disease patient may require pharmacological care and personalized motion, communication support, rehabilitation support, and behavioral therapy. A telemedicine service, in that case, should be personalized. For example, Rare Bone Disease (RBD) patients have several comorbidities associated with other body systems, which requires constant attentive care and cautious multidisciplinary follow-up. However, as we are in the middle of a pandemic, most healthcare workers are busy handling COVID-19 cases in the front line. To manage this emergency, the European Reference Network on Rare Bone Diseases (ERN BOND) brought together 78 experts on RBD, and along with Italian RBD, healthcare professionals created the “COVID-19 Helpline for Rare Bone Diseases” (25, 86). This 24/7 helpline provides high-quality information and recommendations on RBD remotely to patients and healthcare professionals by the RBD experts working in intensive care units or COVID-19 units. Given the convenience of remote consultation, telemedicine can meet people's daily healthcare needs, like cold and fever, without creating pressure at hospitals and timely relieve tensions about the disease.

Telemedicine may not serve the best for the patients if professional and technical characteristics are not maintained. For example, healthcare professionals should answer calls and handle at least 5 years of experience. Even though online services are nowadays app-based, direct telephone communication will allow the patients, especially elderly patients, to communicate rapidly and directly. Most importantly, the service should be in the local language and be available 24/7. According to the local laws, there are different policies and regulations on telemedicine (Table 1) and digitalization in different regions. The concept is new for many of the patients and still evolving by itself as an alternative health consultancy system; both the care providers and the patients need to be aware of their roles and responsibilities to maintain privacy and confidentiality and provide effective feedback to help improve the system. Also, the patients must have the full authority in choosing to participate or to change decisions on whether to continue or not to continue with the service. Moreover, rare disease communities require specialized health professionals to understand better and diagnose their condition promptly.

**Table 1:** Some regulatory and ethical implications of telemedicine in different countries^*^.

**Regulatory and ethical implications**	**Narrative/description**
Legislation and licensing of telehealth products	The telemedicine act from Malaysia and the healthcare services act of Singapore focus on patient safety through proper licensing of medical institutions and healthcare professionals providing telemedicine services (87, 88)
Informed consent and options to choose	Most telemedicine guidelines necessitate the individual's consent, and the patient can change their decision at any time (89)
Privacy, confidentiality, and data security	To ensure data security and confidentiality, Indonesia and Vietnam only allow internet-based registered health facilities for telemedicine service. Indonesia, Malaysia, and Thailand have policies that utilize government information networks for data management and data security. However, according to most guidelines, individual telemedicine providers are responsible for data security (90–94)
Feedback and evaluation	National Telemedicine Guideline of Singapore prioritizes quality improvement activities, cost, accessibility of care, and patient satisfaction. Telemedicine guidelines from Malaysia and Indonesia emphasize communication between doctors and patients to avoid medicolegal consequences (88, 95–97)
Cross border telemedicine	European Union (EU) acts state the right to access to medical treatment in another Member State (Article 1) of European Union (EU), right to access one's written or electronic medical record, (Article 4/2/f), right to be informed about the treatment received, availability, quality, and safety of the service used (Article 4/2/b) (98)
Licensing and qualifications of healthcare professionals	Each national entity in charge of medical practice regulation regulates the qualifications and other legal or ethical aspects of healthcare providers based on its region, including those involved in cross-border telemedicine (98)

* *This table contains only the key data on the regulatory and ethical implications of telemedicine implementation that are most relevant to the COVID-19-related contexts; the references were systemically identified by searching Global Regulations, PubMed, and Google Scholar*.

In a post-pandemic era, the lower-middle-income countries should focus on strengthening the primary health care systems, including trained health professionals who can monitor disease patterns and be alert about the potential outbreaks. Besides that, an instantly accessible trained personnel database and a disease database are also required. For maintaining further emergencies, a predictably safe platform needs to be made where regulatory reviews can be done faster, and massive scale production of therapies, medical supply, and vaccines can be possible. An organized system is necessary for antivirals to screen existing treatments and candidate drugs in a standardized manner. When we return to normal, we must apply what we learned from this pandemic and plan precisely for a dynamic and robust genetic care system for rare disease patients.

## Conclusion

The impact of the COVID-19 pandemic on rare disease communities is asymmetrical in different contexts. While even in a “normal” world, they often face isolation and anxiety due to their uncertain condition and must navigate through several clinicians to obtain the care they need, the additional anxiety due to COVID-19, triggered by the worldwide emergency health protocol and the loaded pressure on health systems, research, development, and the pharma industry, has made the challenges more extravagant than ever. While facing the current challenges, it is essential to keep in mind that access to therapies and continued government and private funding of drug development, translational research, and basic research is crucial to saving rare disease patients' lives. The COVID-19 pandemic experience regarding health emergencies and rare disease management represents the basis for establishing healthcare policies to ensure preparedness for providing adequate care for people with rare diseases.

## Author Contributions

SA conceived the study. SC and SA designed the study, reviewed, and revised the manuscript. SC wrote the draft manuscript. SS helped SC in drafting the manuscript. SA, SC, and SS approved the final version of the manuscript. All authors contributed to the article and approved the submitted version.

## Conflict of Interest

The authors declare that the research was conducted in the absence of any commercial or financial relationships that could be construed as a potential conflict of interest.
